# Impact of Handling Perception and Language Barriers on Virologic Response to Daily Subcutaneous Bulevirtide in Hepatitis D

**DOI:** 10.1111/liv.70404

**Published:** 2025-10-18

**Authors:** Toni Herta, Christopher Dietz‐Fricke, Münevver Demir, Kathrin Sprinzl, Kai‐Henrik Pfeiffer, Julia M. Grottenthaler, Peter Buggisch, Wolfgang Maximilian Kremer, Florian P. Reiter, Andreas Geier, Christoph Schramm, Uta Merle, Christoph Neumann‐Haefelin, Frank Tacke, Thomas Berg, Heiner Wedemeyer, Katja Deterding, Johannes Wiegand

**Affiliations:** ^1^ Department of Hepatology and Gastroenterology, Campus Virchow‐Klinikum (CVK) and Campus Charité Mitte (CCM) Charité – Universitätsmedizin Berlin Berlin Germany; ^2^ Berlin Institute of Health at Charité – Universitätsmedizin Berlin BIH Biomedical Innovation Academy, BIH Charité Clinician Scientist Program Berlin Germany; ^3^ Division of Hepatology, Department of Medicine II Leipzig University Medical Center Leipzig Germany; ^4^ Department of Gastroenterology, Hepatology, Infectious Diseases and Endocrinology Hannover Medical School Hannover Germany; ^5^ Goethe University Frankfurt, University Hospital Germany; ^6^ Department of Internal Medicine B University Hospital Muenster Muenster Germany; ^7^ Department of Gastroenterology, Gastrointestinal Oncology, Hepatology, Infectiology, and Geriatrics University Hospital Tuebingen Tuebingen Germany; ^8^ Ifi‐Institute for Interdisciplinary Medicine Hamburg Germany; ^9^ Department of Internal Medicine I University Medical Center of the Johannes Gutenberg‐University Mainz Germany; ^10^ University Hospital Würzburg Division of Hepatology, Dept. of Medicine II Würzburg Germany; ^11^ Department of Gastroenterology, Hepatology and Transplant Medicine, Medical Faculty University of Duisburg‐Essen Essen Germany; ^12^ Department of Internal Medicine IV University of Heidelberg Heidelberg Germany; ^13^ Department of Medicine II, University Medical Centre Freiburg, Faculty of Medicine University of Freiburg Freiburg Germany; ^14^ Department of Gastroenterology and Hepatology University Hospital Cologne Cologne Germany

**Keywords:** BLV, patient‐reported outcomes, pegylated interferon alfa‐2a, self‐injection, virologic response

## Abstract

**Background:**

Bulevirtide (BLV) is the only approved therapy for chronic hepatitis D (CHD) and compensated liver disease. Daily subcutaneous injection and the need for refrigeration may pose challenges, especially in patients with limited language proficiency.

**Aims:**

Assessment of patient‐reported BLV handling and satisfaction and association with virologic response.

**Methods:**

Patients receiving BLV were assessed using a structured questionnaire on injection preparation, administration, refrigeration, adverse effects, and language proficiency. Virologic response was defined as complete (≥ 2 log10 reduction from baseline or HDV RNA undetectable), intermediate (≥ 1 log10 but < 2 log10 reduction), non‐response (< 1 log10 reduction or increase), or breakthrough (> 1 log10 increase after ≥ 2 log10 reduction).

**Results:**

A total of 115 patients from 30 countries were recruited at 12 German centres. German language skills were rated as good, sufficient, poor or absent in 58%, 25% and 17% of cases. Reported difficulties included injection preparation (17%), administration (25%) and refrigeration (27%). Patients without virologic response or with viral breakthrough reported difficulties with injection administration in 56% compared to 17% with intermediate or complete response (*p* = 0.0002), while no differences were observed regarding preparation or refrigeration. Injection site reactions occurred in 57% of patients. Overall, 82% were satisfied with the practical handling and 94% with the tolerability of BLV. Good language proficiency was associated with greater satisfaction with the practical handling (*p* = 0.0067), which in turn correlated with virologic response (*p* = 0.0001).

**Conclusions:**

BLV administration is generally well manageable. Limited language proficiency and injection difficulties may negatively affect patient satisfaction and potentially influence treatment outcomes.

**Trial Registration:**

German Clinical Trials Registry (DRKS 00033153)

**Summary**

- A relevant proportion of patients with hepatitis D reported practical difficulties with daily subcutaneous bulevirtide self‐injection, particularly injection preparation (17%), injection administration (26%), and maintaining constant refrigeration of the medication (28%).
- Local injection‐site reactions were common (57%) and represented the main issue during administration.
- Importantly, only difficulties with injection administration—but not preparation or cooling—were associated with reduced treatment response, while language skills had no effect on practical challenges or virologic outcome.

## 1 Introduction

Chronic hepatitis delta (CHD) is the most severe form of viral hepatitis, with a higher risk of progression to cirrhosis and hepatocellular carcinoma compared to HBV monoinfection [1, 2, 3]. In July 2023, the sodium taurocholate cotransporting polypeptide inhibitor bulevirtide (BLV) at a daily dosage of 2 mg was granted full authorization by the European Medicines Agency as a therapy for adult patients with CHD and compensated liver disease, representing the only licenced treatment for this condition [4, 5]. Clinical studies have demonstrated multiple benefits: after 48 weeks of monotherapy with 2 mg/day BLV, 45% of patients achieved either undetectable HDV RNA levels or a reduction of at least 2 log10 IU/mL from baseline [6, 7, 8]. Regarding safety, no serious adverse events occurred, and no patient discontinued BLV due to adverse events. Hepatic decompensation or death did not occur [9]. Patient‐reported outcomes related to physical and hepatitis‐specific quality of life under BLV therapy improved compared to untreated patients with CHD, as demonstrated by changes in the EQ‐5D‐3L and Hepatitis Quality of Life Questionnaires, respectively [10]. Similarly encouraging data on antiviral efficacy and clinical safety were demonstrated in various real‐world cohorts after the market approval of BLV [11, 12, 13, 14, 15, 16]. However, in single cases, a re‐increase of HDV RNA serum levels under therapy after initially undetectable HDV RNA levels over a longer period was observed, although patients continued to report full compliance [13, 14].

BLV is a lyophilisate that must be stored under refrigerated conditions (2°C–8°C). Before administration, it requires reconstitution using sterile water for injection. The process involves drawing up the sterile water with one cannula, injecting it into the vial containing the lyophilisate, gently swirling until the substance is fully dissolved, and then drawing up the reconstituted solution with a second sterile cannula for subcutaneous injection. Explaining the preparation of BLV may be challenging, as most patients do not originate from Central Europe and often have a migration background with potential language barriers [17]. We therefore analysed the practical handling and patient satisfaction with daily BLV injections, as well as their potential impact on viral treatment response.

## 2 Patients and Methods

Patients with CHD receiving BLV treatment were prospectively recruited during routine outpatient care between 01/2024 and 02/2025 at 12 centres in Germany. Clinical baseline characteristics, duration of BLV therapy, viral response to treatment, and co‐medications were extracted from patient health records. Virologic response was classified based on HDV RNA kinetics as complete (≥ 2 log10 reduction from baseline or HDV RNA undetectable), intermediate (≥ 1 log10 but < 2 log10 reduction from baseline), non‐response (< 1 log10 reduction from baseline or increase), or breakthrough (> 1 log10 increase after ≥ 2 log10 reduction from baseline) [11].

Handling of daily subcutaneous BLV injections and patient satisfaction were assessed using a project‐specific patient questionnaire, completed by the patient during the outpatient visit (complete questionnaire provided in the Supporting Information). The questionnaire covered:

- Country of birth, native language, German language proficiency,
- Difficulties with reconstituting the medication (e.g., dissolving the powder),
- Difficulties with performing the injection,
- Issues with maintaining the required refrigeration,
- Frequency of missed daily injections,
- Injection site (abdomen or thigh),
- Availability, reading, and perceived helpfulness of the manufacturer's instructional material,
- Local injection site side effects (e.g., redness, hematoma, itching),
- Overall satisfaction with tolerability and administration of the therapy.

## 3 Ethics Statement

Ethical approval for this study (Ethical Committee No. 292/23‐ek) was provided by the Ethical Committee of the University of Leipzig, Leipzig, Germany. The study was conducted in accordance with both the Declarations of Helsinki and Istanbul. Written consent was provided by all patients before study inclusion. The project was registered within the German Clinical Trial Register (DRKS00033153).

## 4 Statistical Analysis

Statistical differences were assessed using the Chi‐square test or Fisher's exact test, depending on applicability. A *p*‐value < 0.05 was considered statistically significant.

If individual data were not available for specific items, the corresponding denominator of the cohort is mentioned in the text. Statistical analyses were performed using software from the R Development Core Team (2011), version 4.2.0.

## 5 Results

### 5.1 Baseline Characteristics of the Cohort

A cohort of 115 patients was prospectively recruited at 12 German hepatology centres over a period of 14 months. Baseline characteristics of the cohort are summarised in Table 1. The median recruitment rate per centre was 5.5 (range: 3–34) cases. Of the 115 patients, 74 (64%) were male, with a median age of 48 years (range: 25–82). Cirrhosis was present in 56% (63/112) of cases. Child‐Pugh classification was available in 48 of these 63 cases, with the following distribution: class A in 92% (44/48), class B in 6% (3/48), and class C in 2% (1/48).

**TABLE 1:** Baseline characteristics of the study cohort.

Characteristic		*n*/*N* (%)
Gender	Male	74/115 (64%)
	Female	41/115 (36%)
BLV treatment duration	> 6 months	94/108 (87%)
	3–6 months	8/108 (7%)
	1–3 months	6/108 (6%)
Language skills	First language	17/109 (16%)
	Good	66/1114 (58%)
	Sufficient	28/114 (25%)
	Poor	15/114 (13%)
	Absent	5/114 (4%)

*Note:* The denominator for each item indicates the number of patients with available data.

Abbreviation: BLV, bulevirtide.

BLV treatment duration was > 6 months in 87% (94/108). An ongoing combination therapy with pegylated interferon alfa was used in 7% (6/88) of patients. 91% (86/95) of individuals received concomitant nucleoside/nucleotide analogues.

Hepatitis D related virologic response was complete in 46% (53/114), intermediate in 32% (36/114), and absent in 14% (16/114). A viral breakthrough was observed in 8% (9/114). A prior treatment with pegylated interferon alfa was reported by 47% (50/107).

Patients originated from 30 different countries, most frequently from Russia (17% [18/109]), Kazakhstan (11% [12/109]), Moldova (11% [12/109]), and Turkey (9% [10/109]). Only one individual indicated Germany as the country of origin. Nevertheless, 16% (17/109) of patients identified German as their first language, while Russian was the most frequently spoken native language (30% [33/109]). German language skills were rated as very good or good (hereafter referred to as ‘good’) by 58% (66/114), sufficient by 25% (28/114), poor by 13% (15/114), and absent by 4% (5/114). The manufacturer's instruction for using BLV was provided in a language understood by 82% (94/115); 82% (84/102) read it, and 83% (79/95) found it helpful.

### 5.2 Practical Handling of Daily BLV Injections

**FIGURE 1:**
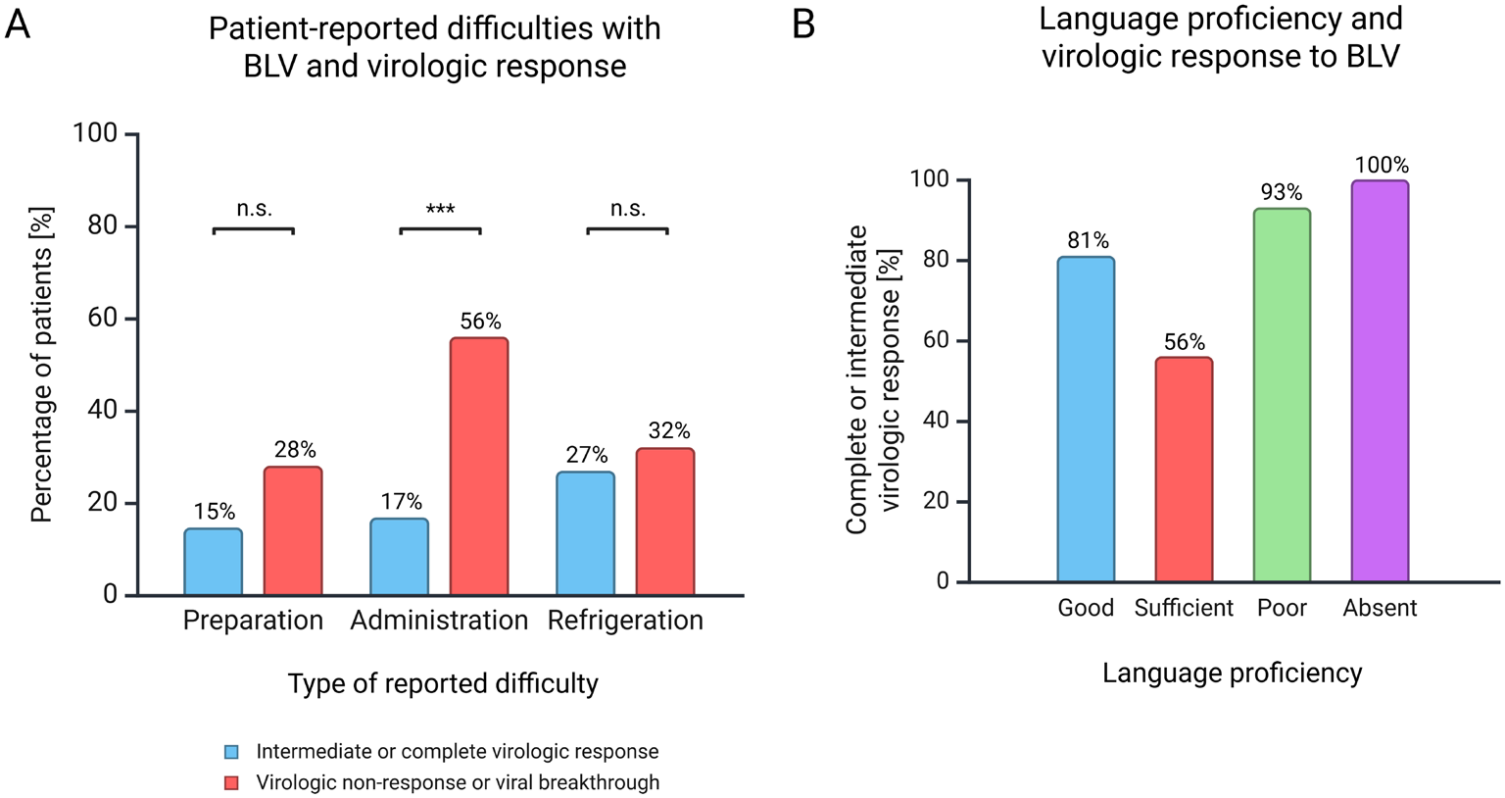
Impact of reported difficulties with BLV injection (A) and language proficiency (B) on virologic response to BLV treatment. BLV, bulevirtide; n.s., not significant. Statistics: Chi‐squared test, ***p < 0.001.

Difficulties with the preparation of the injection were reported by 17% (20/115), difficulties with the administration of the injection by 25% (29/115), and difficulties with maintaining uninterrupted cooling by 28% (32/115) (Table 2). German language skills, prior treatment with pegylated interferon alfa, or BLV treatment duration had no influence on the frequency of these difficulties. Difficulties with injection preparation or uninterrupted cooling had no influence on the virologic response. However, patients without a virologic response or with a viral breakthrough experienced more frequent difficulties with injection administration compared to patients with complete or intermediate virologic response (56% [14/25] vs. 17% [15/89], *p* = 0.0002) (Figure 1A).

**TABLE 2:** Frequency of practical difficulties with daily BLV injections.

		Difficulties with BLV preparation	Difficulties with BLV injection	Difficulties with BLV cooling
Overall cohort (*n* = 115)		20 (17%)	29 (26%)	32 (28%)
Frequency of occurrence in affected individuals	Rarely	16/20 (80%)	22/29 (76%)	26/32 (81%)
	Frequently	4/20 (20%)	6/29 (21%)	5/32 (16%)
	Mostly	0/20 (0%)	1/29 (3%)	1/32 (3%)

Abbreviation: BLV, bulevirtide.

Patients rated their satisfaction with the practical handling of the daily BLV injection as very satisfied or satisfied (hereafter referred to as ‘satisfied’) in 82% (94/115), indifferent in 9% (10/115), or dissatisfied or very dissatisfied (hereafter referred to as ‘dissatisfied’) in 10% (11/115). Patients who were satisfied with the practical handling reported more often good German language skills compared to indifferent or dissatisfied patients (64% [60/94] vs. 29% [6/21], *p* = 0.0067). Prior or ongoing pegylated interferon alfa therapy had no impact on patient satisfaction with the practical handling of BLV injections. Satisfied patients showed a higher rate of complete or intermediate virologic response compared to indifferent or dissatisfied patients (86% [80/93] vs. 43% [9/21], *p* = 0.0001). However, proficiency in the German language had no impact on virologic response (Figure 1B).

25% (29/115) of the patients indicated that they occasionally forgot the injection, and 2% (2/115) indicated that they forgot it frequently. Occasional forgetting of the injection had no impact on the virologic response to BLV therapy in our cohort. The influence of frequently missed injections on treatment response could not be evaluated due to the low number of such cases.

### 5.3 Tolerability of Daily BLV Injections

BLV was administered into the subcutaneous abdominal fat by 71% (79/112), into the thigh by 10% (11/112), or alternately at both sites by 20% (22/112). Injection site reactions occurred in 57% (66/115) of patients, in descending order of frequency: hematoma (41% [47/115]), redness (35% [40/115]), local itching (24% [28/115]), bleeding (4% [5/115]), pain (4% [4/115]), swelling (4% [4/115]), and foreign body sensation (2% [2/115]) (Table 3). All local adverse events were mild to moderate in severity; none were severe. Both the frequency and severity of injection site reactions did not differ between patients with cirrhosis and those without cirrhosis. However, patients who reported difficulties with injection administration experienced injection site reactions more frequently than those without such difficulties (79% [23/29] vs. 50% [43/86], *p* = 0.009).

**TABLE 3:** Injection site reactions to BLV injections.

		Hematoma	Redness	Local itching	Bleeding	Pain	Swelling	Foreign body sensation
Overall cohort (*n* = 115)		47 (41%)	40 (35%)	28 (24.3%)	5 (4%)	4 (4%)	4 (3.5%)	2 (2%)
Frequency of occurrence in affected individuals	Rarely	33/47 (70%)	29/40 (73%)	19/28 (67.8%)	4 (80%)	3 (75%)	3 (75%)	0 (0%)
	Frequently	14/47 (30%)	11/40 (28%)	8/28 (28.5%)	1 (20%)	1 (25%)	1 (25%)	2 (100%)
	Mostly	0/47 (0%)	0/40 (0%)	1/28 (3.5%)	0 (0%)	0 (0%)	0 (0%)	0 (0%)

Abbreviation: BLV, bulevirtide.

Patients rated their satisfaction with the tolerability of BLV injections predominantly positive: 94% (108/115) as very satisfied or satisfied (hereafter referred to as ‘satisfied’), 4% (5/115) as indifferent, and 2% (2/115) as dissatisfied or very dissatisfied (hereafter referred to as ‘dissatisfied’). Satisfaction with BLV tolerability was similarly high amongst patients with or without cirrhosis, as well as amongst those with or without prior pegylated interferon alfa therapy. Ongoing co‐medication with pegylated interferon alfa also had no impact on patient‐rated satisfaction with the tolerability of BLV injections. Patient satisfaction with BLV tolerability was not associated with virologic response.

## 6 Discussion

In this multicentre real‐world cohort of 115 individuals with CHD, we present a cross‐sectional systematic analysis of patient‐reported handling, tolerability, and satisfaction with daily BLV injections, and their potential influence on virologic response to treatment.

Our findings indicate that, despite the need for daily subcutaneous self‐administration, overall patient satisfaction with the practical handling of BLV is high. Language barriers affect a substantial proportion of patients. Those with good language skills reported greater satisfaction with the practical handling of daily injections. However, it must be considered that language skills were assessed by categorical self‐report and not by objective language certificates, which may introduce a self‐reporting bias and limit the conclusions related to language in this manuscript.

The overall positive patient satisfaction with the daily injection aligns with previously reported high acceptance of other long‐term therapies requiring regular subcutaneous self‐administration [18, 19]. Acceptance is particularly high when patients understand the necessity of the treatment aiming at reducing the long‐term risk of clinical events, especially in asymptomatic conditions where the injection does not result in immediate, perceptible symptom relief [19, 20]. Better patient education about the rationale and long‐term benefits of treatment may have contributed to the greater satisfaction observed amongst patients with stronger language skills, rather than simply clearer instructions on injection technique. These technical aspects are already covered by the manufacturer's instructions, which most patients received, read, and found helpful, as they were provided in a language they understood. Additionally, reported difficulties with preparation, administration, or refrigeration of BLV occurred independently of language skills. We further observed that greater satisfaction with the practical handling was associated with higher rates of complete or intermediate virologic response. However, this observation should be interpreted with caution, as patients are typically aware of their treatment outcomes. Treatment efficacy influences patient acceptance and attitudes towards long‐term medication use [19, 20, 21]. It is therefore likely that patients with a favourable response perceive both their therapy and its practical handling more positively, rather than the reverse.

Despite overall high patient satisfaction with the practical handling of BLV, a relevant proportion of patients reported difficulties with injection preparation (17%), administration (25%), or maintaining uninterrupted cooling (28%). Based on practical experience, examples of such difficulties are uncertainty about whether the medication is fully dissolved (preparation), injecting too shallowly or too deeply (administration), or ensuring proper refrigeration during travel (cooling) [22, 23, 24, 25]. We observed that difficulties with the administration of the injection, but not with preparation or cooling, may affect the virologic response to BLV treatment. Difficulties in self‐administration of daily injections can result in poor treatment adherence [20, 26]. Indeed, 25% of patients reported occasionally omitting the injection. However, this did not affect the virologic response. On the other hand, too shallow injection with deposition of BLV in the dermis or epidermis rather than the subcutaneous tissue layer may impair absorption [27], which could potentially result in reduced treatment effectiveness. Since too shallow deposition may also increase local reactions [27, 28], this could explain why patients reported administration as difficult, as they are usually unaware of injection errors unless reactions occur. It must be noted, however, that our assessment is solely based on self‐reported data by patients, which are difficult to objectify. Moreover, it was not assessed which specific difficulty occurred. Thus, whether administration difficulties with BLV such as too shallow injection actually contribute to treatment outcomes such as viral breakthrough despite continuously reported compliance in individual cases [13, 14] remains hypothetical. Nevertheless, in clinical practise, particular emphasis should be placed on identifying and correcting injection administration issues. For example, performing the injection together on a dummy in the clinic can effectively help identify and resolve such difficulties [20].

Injection‐site reactions were frequently reported in our cohort and occurred more often than in the pooled safety analysis of the phase 2 and 3 BLV trials [9]. 57% in our cohort reported injection‐site reactions, primarily hematoma, redness, and local itching, compared to 16% in the pooled clinical trial analysis [9]. Difficulties with injection administration might contribute to this difference, as discussed above. However, as in the clinical trials, all injection‐site reactions were mild to moderate and self‐limiting within a few days at most, and did not negatively impact the overall positive patient assessment of BLV tolerability. Therefore, BLV represents a safe and well‐tolerated therapy for patients with CHD. This contrasts with pegylated interferon alfa‐2a, which was associated with treatment interruption due to serious adverse events in 3 of 13 individuals during long‐term real‐life treatment, or with premature discontinuation before study week 120 in 22 of 120 individuals in the HIDIT‐II study [29, 30].

It is important to emphasise that both overall satisfaction with BLV tolerability and the local tolerability of BLV injections were not different between patients with and without cirrhosis. This supports, also from a patient's perspective, that BLV can be safely used in patients with cirrhosis [12, 16, 31, 32].

In summary, our data indicate that the daily injection of BLV is generally well manageable for patients. Language proficiency influences patient satisfaction with the practical handling of BLV. Difficulties with injection administration appear to be particularly relevant and may influence both the occurrence of local injection‐site reactions and virologic response. These insights underscore the importance of patient education and practical support to optimise therapeutic benefit.

## Author Contributions

T.H. and J.W.: conceptualisation. T.H., C.D.‐F., K.D., H.W. and J.W.: methodology. All authors: Data acquisition. T.H. and J.W.: formal analysis. T.H. and J.W.: Writing – original draft preparation. All authors: writing – review and editing.

## Conflicts of Interest

T.H.: consulting fees of Albireo (Ipsen), Advanz, advisory board member for Advanz, Gilead, Amgen. C.D.‐F.: Receipt of grants/research and travel support: Gilead. M.D.: lecturer and advisory board member for Gilead, Abbvie, Falk, Advanz, Ipsen, Merz, Boehringer, GSK, Astra Zeneca, Sanofi; receipt of grants research supports from Merz, Echosens. K.S.: Lecture fees: AbbVie, MSD, Gilead, Chemomab; Consulting: Gilead, Ipsen; Project funding: Gilead; Travel grants: Abbvie, Gilead. P.B.: lecturer and advisory board member for Abbvie, Astra Zeneca, Advanz, Falk, Gilead, Ipsen, Mirum, NovoNordisk, Norgine, Orphalan, Pfizer, Roche, Sanofi, Sobi, Univar. A.G.: Advisor and lecturer for AbbVie, Advanz, Albireo, Alexion, AstraZeneca, Bayer, BMS, Boehringer, Burgerstein, CSL Behring, Eisai, Falk, Gilead, Heel, Intercept, Ipsen, Madrigal, Merz, MSD, Novartis, NovoNordisk, Orphalan, Pfizer, Roche, Sanofi‐Aventis; research support from: Intercept and Falk (*NAFLD CSG*), Boehringer, Novartis. U.M.: Consultant or Speaker Honorarium: CytoSorbents, Falk, Gilead, Microbiotica, Univar. C.N.H.: Speakers bureau: Abbvie, Falk Foundation, Gilead. F.T.: Research grant: MSD, Gilead, Agomab. Speakers bureau: Gilead, AbbVie, Falk, MSD. Consultant: Boehringer, Madrigal, GSK, Ipsen, Mirum, AstraZeneca, Gilead, AbbVie, BMS, Pfizer, Novartis, Novo Nordisk, Sanofi. T.B.: Receipt of grants/research supports: Abbvie, BMS, Gilead, MSD/Merck, Humedics, Intercept, Merz, Norgine, Novartis, Orphalan, Sequana Medical; Receipt of honoraria or consultation fees/advisory board: Abbvie, Alexion, Albireo, Bayer, Gilead, GSK, Eisai, Enyo Pharma, HepaRegeniX GmbH, Humedics, Intercept, Ipsen, Janssen, MSD/Merck, Novartis, Orphalan, Roche, Sequana Medical, SIRTEX, SOBI, and Shionogi; Participation in a company‐sponsored speaker's bureau: Abbvie, Advance Pharma, Alexion, Albireo, Bayer, Gilead, Eisai, Falk Foundation, Intercept, Ipsen, Janssen, MedUpdate GmbH, MSD/Merck, Novartis, Orphalan, Sequana Medical, SIRTEX, and SOBI. H.W.: lecturer, consultancy and advisory board member for Falk Pharma, Gilead Sciences, MSD, Biotest; lecturer for BioMarin, CSL Behring, Falk Foundation, Qlink; consultancy and advisory board member for Abbott, Albireo, AstraZeneca, Atea Pharmaceuticals, Bristol‐Myers Squibb, Hoffmann‐La Roche, GlaxoSmithKline, Janssen, Lilly, Mirum, Orphalan, Pfizer, Roche Diagnostics, Roche Pharma, Sobi—Swedish Orphan Biovitrum, Takeda, Vir Biotechnology; Grants/research supports from Biotest, Abbott. K.D.: Lecture fees, travel grants from Gilead and AbbVie; research grant and advisory board from Gilead. J.W.: lecturer and advisory board member for Abbvie, Intercept/Advanz Pharma, GSK, Ipsen, Gilead Sciences, NovoNordisk; receipt of grants/research supports from Abbvie, Gilead Sciences, NovoNordisk. K.‐H.P., J.M.G., W.M.K., F.P.R., C.S.: Nothing to disclose.

## Supporting information


**Data S1:** liv70404‐sup‐0001‐supinfo01.pdf.

**Data S2:** liv70404‐sup‐0002‐supinfo02.pdf.

## Data Availability

The data that support the findings of this study are available from the corresponding author upon reasonable request.
