# Supplementary material for: Impact of Handling Perception and Language Barriers on Virologic Response to Daily Subcutaneous Bulevirtide in Hepatitis D
Source: Liver Int. 2025 Oct 18;45(11):e70404. doi: 10.1111/liv.70404 (PMC12535276; doi:10.1111/liv.70404)
Supplement: Supplementary file 1 — Data S1: liv70404‐sup‐0001‐supinfo01.pdf. [file LIV-45-0-s002.pdf]

Professor Dr. Johannes Wiegand und Dr. Toni Herta
Universitätsklinikum Leipzig, Medizinische Klinik II

Datenerfassungsfragebogen

Studie: „*Untersuchung der Auswirkung der Patientenzufriedenheit mit der subkutanen Injektion von Bulevirtid auf das Therapieansprechen bei Hepatitis B/D-Koinfektion*."

Patienten-ID (Hepcludex® Real-World Kohorte MHH Hannover): __________

Datum: __________

Alter des Patienten: __________ (Jahre)

Geschlecht: □ Männlich □ Weiblich □ Divers

Geburtsland: __________

Leberzirrhose: □ Ja □ Nein

Dauer der Behandlung: __________ (Wochen)

Virologisches Ansprechen:
□ Vollständig
□ Teilweise
□ Nicht-Ansprechen
□ Viraler Durchbruch

Biochemisches Ansprechen:
□ Vollständig
□ Teilweise
□ Nicht-Ansprechen
