# Supplementary material for: Impact of Handling Perception and Language Barriers on Virologic Response to Daily Subcutaneous Bulevirtide in Hepatitis D
Source: Liver Int. 2025 Oct 18;45(11):e70404. doi: 10.1111/liv.70404 (PMC12535276; doi:10.1111/liv.70404)
Supplement: Supplementary file 2 — Data S2: liv70404‐sup‐0002‐supinfo02.pdf. [file LIV-45-0-s001.pdf]

Professor Dr. Johannes Wiegand und Dr. Toni Herta
Universitätsklinikum Leipzig, Medizinische Klinik II
Fragebogen Version 1 (26.06.2023)

Fragebogen zur Durchführung der s.c. Injektion von Hepcludex®

Patienten-ID (Hepcludex® Real-World Kohorte MHH Hannover): __________

Datum: __________

Alter des Patienten: __________ (Jahre)

Geschlecht: □ Männlich □ Weiblich □ Divers

Geburtsland: __________

Muttersprache: __________

Deutschkenntnisse: □ Sehr gut □ Gut □ Ausreichend □ Schlecht □ Keine

Wurde bereits früher (Peg-)Interferon injiziert? □ ja □ nein

Dauer der Behandlung mit Hepcludex®: □ < 4 Wochen □ 1-3 Monate □ 3-6 Monate □ >6 Monate

Treten Schwierigkeiten bei der Vorbereitung der Injektion auf (Auflösen des Pulvers etc.)?

□ Nie □ Selten □ Häufig □ Immer

Treten Schwierigkeiten bei der Durchführung der Injektion auf?

□ Nie □ Selten □ Häufig □ Immer

Treten Schwierigkeiten bei der Kühlung von Hepcludex® auf?

□ Nie □ Selten □ Häufig □ Immer

Wie häufig vergessen Sie die tägliche Injektion von Hepcludex?

□ Nie □ Selten □ Häufig □ Immer

Wo wird die Injektion appliziert? □ Bauch □ Oberschenkel

Wurde das Informationsmaterial des Herstellers zur Vorbereitung und Durchführung der Injektion von Hepcludex® zur Verfügung gestellt? □ ja □ nein

Wenn ja, wurde das Informationsmaterial des Herstellers gelesen? □ ja □ nein

Wenn ja, wurde das Informationsmaterial als hilfreich empfunden? □ ja □ nein

Treten Nebenwirkungen an der Injektionsstelle auf? □ ja □ nein

Wenn ja (bitte für zutreffende Nebenwirkung ankreuzen):

Nebenwirkung	Häufigkeit	Schweregrad
Rötung	□ Nie<br>□ Selten<br>□ Häufig<br>□ Immer	□ Gering<br>□ Mittelgradig<br>□ Schwerwiegend
Hämatom	□ Nie<br>□ Selten<br>□ Häufig<br>□ Immer	□ Gering<br>□ Mittelgradig<br>□ Schwerwiegend
Juckreiz	□ Nie<br>□ Selten<br>□ Häufig<br>□ Immer	□ Gering<br>□ Mittelgradig<br>□ Schwerwiegend
Andere __________	□ Nie<br>□ Selten<br>□ Häufig<br>□ Immer	□ Gering<br>□ Mittelgradig<br>□ Schwerwiegend

Wie zufrieden sind Sie mit der Verträglichkeit der Hepcludex® Therapie?

□ Sehr zufrieden □ Zufrieden □ Egal □ Unzufrieden □ Sehr unzufrieden

Wie zufrieden sind Sie insgesamt mit der Durchführung der Hepcludex® Therapie?

□ Sehr zufrieden □ Zufrieden □ Egal □ Unzufrieden □ Sehr unzufrieden
